# Why psychiatry is different - challenges and difficulties in managing a nosocomial outbreak of coronavirus disease (COVID-19) in hospital care

**DOI:** 10.1186/s13756-020-00853-z

**Published:** 2020-12-01

**Authors:** J. J. E. Rovers, L. S. van de Linde, N. Kenters, E. M. Bisseling, D. F. Nieuwenhuijse, B. B. Oude Munnink, A. Voss, M. Nabuurs-Franssen

**Affiliations:** 1 Department of Psychiatry, Canisius-Wilhelmina Hospital, Weg door Jonkerbos 100, 6534 SZ Nijmegen, The Netherlands; 2 Department of Medical Microbiology and Infectious Diseases, Canisius-Wilhelmina Hospital, Nijmegen, The Netherlands; 3 Department of Medical Microbiology, Radboud University Medical Centre, Nijmegen, The Netherlands; 4 Department of Viroscience, Erasmus University Medical Centre, Rotterdam, The Netherlands

**Keywords:** COVID-19, Hospital psychiatry, Nosocomial outbreak, Hospital outbreak management

## Abstract

**Objective:**

Coronavirus disease (COVID-19) was officially declared a pandemic in March 2020. Many cases of COVID-19 are nosocomial, but to the best of our knowledge, no nosocomial outbreaks on psychiatric departments of severe acute respiratory syndrome coronavirus 2 (SARS-CoV-2) have been reported in Europe. The different nature of psychiatry makes outbreak management more difficult. This study determines which psychiatry specific factors contributed to a nosocomial outbreak taking place in a psychiatric department. This will provide possible interventions in future outbreak management.

**Method:**

A case series describing a nosocomial outbreak in a psychiatric department of an acute care hospital in the Netherlands between March 13, 2020 and April, 14 2020. The outbreak was analyzed by combining data from standardized interviews, polymerase chain reaction (PCR) tests and whole genome sequencing (WGS).

**Results:**

The nosocomial outbreak in which 43% of staff of the psychiatric department and 19% of admitted patients were involved, was caused by healthcare worker (HCW)-to-HCW transmissions, as well as patient-to-HCW-to-patient transmission. We identified four aspects associated with the mental health care system which might have made our department more susceptible to an outbreak.

**Conclusions:**

Infection control measures designed for hospitals are not directly applicable to psychiatric departments. Psychiatric patients should be considered a high-risk group for infectious diseases and customized measures should be designed and implemented. Extra attention for psychiatric departments is necessary during a pandemic as psychiatric HCWs are less familiar with outbreak management. Clear communication and governance is crucial in correctly implementing these measures.

## Introduction

In December 2019, an unknown coronavirus was linked to a surge in patients with fever and pneumonia in China. The causative agent was identified as severe acute respiratory syndrome coronavirus-2 (SARS-CoV-2) and the resulting disease was named coronavirus disease (COVID-19). On March 11, the World Health Organization (WHO) declared COVID-19 a pandemic. It is thought that ongoing surveillance of SARS-CoV-2 is warranted as a re-emergence of the disease could still occur over the following years [1]. Consequently, prolonged continuation of social distancing policies might be needed for the foreseeable future to avoid an overburdening of the health care system [1].

SARS-CoV-2 is a betacoronavirus related to severe acute respiratory syndrome (SARS) virus [2, 3]. Routes of transmission include person-to-person spread via respiratory droplets and by touching contaminated objects [4, 5]. The mean incubation interval is 5.2 days, with 95% of the cases occurring in maximum 12.5 days following infection [6]. Evidence suggests that transmission might occur more frequently at the onset of infection [7]. The initial presentation consisted of fever, fatigue and a dry cough, but other, milder symptoms have also now been documented [8]. The majority of infections are mild (81%) and self-limiting. Severe and critical infections account for about 14 and 5% of cases, and may be complicated by acute respiratory distress syndrome (ARDS) and multiple organ failure [9]. Since the clinical manifestation of infection ranges from severe to asymptomatic, there have been concerns that transmission might be able to occur due to unquarantined individuals and healthcare workers (HCWs) with no or few symptoms going unnoticed [8].

Hospital outbreaks are either nosocomial or community-acquired, can place a heavy burden on already overwhelmed health care systems [10, 11], and pose risks to both patients and medical staff. One study found that in 41% of admitted patients with COVID-19, nosocomial transmission was the suspected source of infection, whereas another study found that HCWs accounted for 3.8% of total cases [12, 13]. It is thought that inadequate personal protection, insufficient understanding of COVID-19 and infection and prevention control, prolonged exposure to large groups of infected patients and lack of personal protective equipment (PPE) contributed to an increase in nosocomial infections in HCWs [14, 15]. It is discussed that inpatient psychiatric care faces specific challenges, including the risk for nosocomial outbreaks [16].

To the best of our knowledge, no nosocomial outbreaks on psychiatric departments have yet been described in Western countries. In March 2020, the department of psychiatry of the Canisius-Wilhelmina Hospital (CWZ) in Nijmegen reported a sudden increase in SARS-CoV-2 infections amongst both admitted psychiatric patients and medical staff. A nosocomial outbreak of COVID-19 was declared on March 23. We conducted a case series to identify the weaknesses and risk factors within the mental health care system, which may contribute to nosocomial outbreaks in psychiatry.

## Methods

### Setting

The nosocomial outbreak occurred in the CWZ in Nijmegen, The Netherlands. The CWZ is a 480-bed acute care hospital. The outbreak was limited to the psychiatric department, which consists of an 18-bedded psychiatric ward, an outpatient clinic and a consultation-liaison unit, and currently employs 70 HCWs.

On March 1, 2020, the CWZ implemented precautionary measures to prevent further spread of SARS-CoV-2 infections. General measures included reverse transcription polymerase chain reaction (RT-PCR) testing of all symptomatic patients and HCWs, stringent practice of hand hygiene, social distancing and (contact and droplet) isolation of (suspected) SARS-CoV-2 patients. Additional measures aimed at employees consisted of self-reporting of symptoms and measuring body temperature twice daily. HCWs with COVID-19 symptoms and/or fever, were tested. Following the test they were not allowed to return to work until they had been symptom-free for 72 h. Employees without a fever and without specific symptoms were not tested and were allowed to work using surgical masks.

### Case identification

The identification of suspected employees relied on them reporting their signs and symptoms to the occupational health service (OHS). Based on the probability of infection, nasopharyngeal swabs (PCR) for SARS-CoV-2 were performed. Suspected patients were identified by the attending psychiatrist, and were tested accordingly. We defined confirmed cases based on a positive test result. Suspected cases who tested negatively were also included, as negative PCR tests are known to be false negative, due to sampling errors, premature or delayed testing or an inadequate swab technique. Verbal informed consent was obtained from all HCWs and two out of three patients, as the third patient was deceased at the study’s onset. All was carried out accordingly to Dutch law and the code of conduct in scientific research.

### Design

A standardized questionnaire was carried out amongst confirmed and suspected cases in order to collect data. The signs and symptoms in our questionnaire were based on a similar case questionnaire used by the ministerial department of health of the New South Wales Government in Australia [17]. Questions about employees’ working schedules were added. The collected data included demographic information, signs and symptoms and their date of onset, SARS-CoV-2 test results, risk factors and clinical outcome. In order to reconstruct a suspected chain of transmission, we interviewed the confirmed cases, the attending psychiatrist responsible for the psychiatric ward and the head of the psychiatric department. Additionally, we reviewed employees’ working schedules and patients’ medical records.

### Diagnostics

RT- PCR targeting of the E-gene was performed on nasopharyngeal swabs from patients and HCWs. Ribonucleic acid (RNA) was isolated using the Roche MagnaPure 96 in combination with the master mix (Taqman Fast Virus 1-Step master mix). PCR was performed by using primers and probes as described by Corman et al. [18]. WGS was carried out on positive samples according to the methods described by Oude Munnink et al. [19]. In short, 87 overlapping amplicons spanning the entire genome were generated in two different reactions, after which they were sequenced on the Nanopore sequencing platform.

### Minimum spanning tree

A minimum spanning tree (MST) was made using the generated SARS-CoV-2 genome sequences. Sequence distance was calculated based on absolute nucleotide and gap-block distance using the Analysis of Phylogenetics and Evolution (APE) R package [20]. The MST was subsequently derived from the all-vs-all sequence distance network using the igraph R package [21]. The visualization was generated using the visNetwork R package.

## Results

A total of 33 cases were identified between March 13, 2020 and April 14, 2020, of which 15 cases were confirmed and 18 cases were suspected. The confirmed cases consisted of 3 patients and 12 HCWs, amounting to 19% of total admitted patients and 17% of the total staff number. The remainder of suspected cases (*n* = 18) consisted of HCWs only and amounted to 43% of the total staff number. No other admitted patients were considered suspected cases. The mean age of all cases was 43.3 years, 61% were female and HCWs accounted for 91% of cases (Table 1). One patient died due to COVID-19, whereas no fatalities occurred in HCWs.

**Table 1:** Characteristics of confirmed and suspected cases in the nosocomial outbreak of SARS-CoV-2

Characteristic	Number and percentage of cases (***n*** = 33)
**Sex**	
Male Female	13 (39%) 20 (61%)
**Age**	
Mean (year)	43.3 (range 21–77)
**Risk factors**	
COPD Diabetes Cardiovascular disease Cancer	4 (12%) 2 (6%) 2 (6%) 1 (3%)
**Status of case**	
Healthcare worker Patient	30 (91%) 3 (9%)
**PCR for SARS-CoV-2**	
Positive Negative Not tested	15 (45%) 17 (52%) 1 (3%)
**Outcome**	
Alive Deceased	32 (97%) 1 (3%)

### Outbreak timeline

**Fig. 1:**
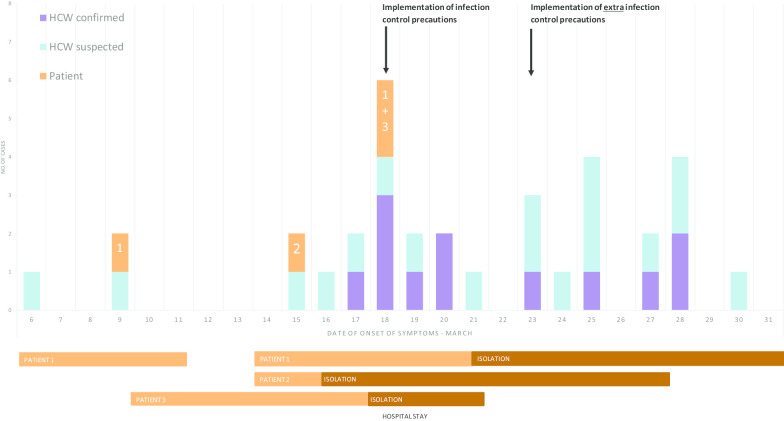
Suspected and confirmed cases of HCWs and patients with SARS-CoV-2 by date of onset of symptoms from March 6 to March 31, 2020. Duration of hospital admission and isolation of patients 1–3 are visualized accordingly

On March 9, 2020, the first patient and HCW started showing symptoms of COVID-19 (see Fig. 1). The patient was tested within 48 h after onset of symptoms and was found to be negative for SARS-CoV-2. The HCW was not tested for COVID-19 and continued to work without PPE, thereby unwittingly ignoring national guidelines.

On March 15, the second patient started showing symptoms (Fig. 1). The patient had been admitted to the psychiatric department the day before. She was brought in by her son who was not feeling well at the time and developed a fever the following day. The patient was in a catatonic state and therefore adequate screening for COVID-19 was not possible. On March 17, 2020, the patient also developed fever, was directly placed in contact droplet isolation and was subsequently tested positive for COVID-19 the day after. Infection control was consulted to discuss the first confirmed case on the psychiatric department. Infection control found that the hospital wide infection prevention policy was only partially implemented on the psychiatric ward. The implemented measures of social distancing between patients and HCWs, twice daily measuring of body temperature of patients and HCWs, and the adequate use of PPE by HCWs when tending to patients, were not followed satisfactorily. Additionally, group therapy for admitted patients continued to take place.

On March 18, when reporting of symptoms was strictly implemented for employees and patients, the first employees of the psychiatric department tested positive for COVID-19. These employees had been in close contact to the second patient without using PPE. Retrospectively, eight employees and two patients showed COVID-19 symptoms in the period from March 17 to March 20, 2020. Not every employee reporting loss of smell and/or taste was tested, as this was not classified as a symptom of COVID-19 at the time. Due to conflicting advice from the OHS, multiple HCWs continued to work with symptoms. Additionally, a third patient started showing COVID-19 symptoms and, after an initial symptom free interval, the first patient developed new symptoms. Both tested positive on March 19 and March 23 respectively.

On March 23, additional infection control measures were implemented on the psychiatric department to limit further transmission. These measures included transferring SARS-CoV-2 positive patients to designated hospital wards, classifying all remaining patients and personnel as SARS-CoV-2 suspected, measuring body temperature of patients thrice daily and actively enquiring about COVID-19 symptoms, demanding all employees wear surgical masks at all times, reducing bed-capacity to 8 beds, reducing working staff, training of staff in the use of PPE and implementing stricter compliance to the hospital wide COVID-19 policies (e.g. social distancing, hand hygiene, reporting of symptoms and measuring body temperature by personnel twice daily).

On March 31, no new patients were discovered and the outbreak was considered to be contained. Staff members were obligated to wear a surgical mask until April 14. All other implemented measures remained in place.

## Outbreak interpretation

The epidemic curve does not show a clear index case. Due to insufficient testing of both symptomatic and asymptomatic HCWs, and the absence of serological tests for the identification of previous infections, the results are not conclusive. Furthermore, one patient was tested within 48 h after onset of symptoms and was not retested. Current policies dictate that retesting is necessary, since the first test may be false negative.

**Fig. 2:**
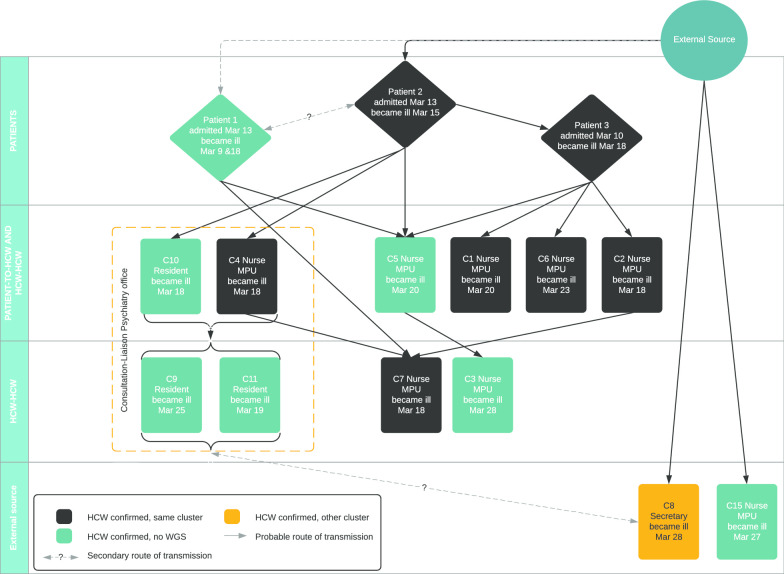
Probable route of transmission of 15 confirmed cases of SARS-CoV-2 in the nosocomial outbreak, linked to each other based on probability, PCR results and WGS

Out of the 15 samples of confirmed cases, seven samples, belonging to five HCWs and two patients, were subjected to WGS. Nine samples had a cycle threshold (CT) value higher than 30, thereby making WGS impossible. The minimum spanning tree (MST, Additional file 1) revealed that four out of five HCWs’ samples and both patients’ samples were identical and one HCW‘s sample was only separated by a single nucleotide. Based on the combined evidence of the probability of exposure, PCR positive test results and WGS, it is evident that transmission occurred between patients and HCWs. Furthermore, three confirmed HCWs had no contact with confirmed patients, but did have contact with confirmed colleagues, thereby implying that transmission between HCWs took place as well. In Fig. 2 the likeliest route of transmission is visualized. Only confirmed cases were included. One HCW (depicted as C15 in Fig. 2) had a husband with COVID-19 symptoms, who worked at another healthcare institution experiencing an outbreak, and another HCW (depicted as C8) belonged to a different cluster. Therefore, it is possible that multiple introductions occurred on the department.

## Discussion

The aim of our research was to identify the weaknesses and risk factors within psychiatry which may contribute to nosocomial outbreaks. We identified four aspects associated with psychiatry which made our department more susceptible to an outbreak, and from which valuable lessons could be learned in order to prevent outbreaks in the future.

### The psychiatric population

Psychiatric patients are more at risk during a pandemic. Firstly, patients with severe mental illness are more susceptible to pulmonary infections, whereas patients with depressive disorders are more at risk of developing infectious diseases in general [22–24]. Secondly, the recognition and management of physical disease in psychiatric patients is often suboptimal in comparison with the general population [25, 26]. Psychiatrists often consider it their primary task to exclusively treat psychiatric illnesses, thereby possibly overlooking signs of physical disease [27]. Furthermore, patients with severe mental illness are often unaware of their physical symptoms due to cognitive deficits or reduced pain sensitivity, and frequently have trouble communicating [26]. For instance, our second patient’s presentation of symptoms was severely impaired due to her catatonic state. Thirdly, mental illnesses and psychiatric medications tend to negatively influence patients’ capabilities of comprehending and following instructions [26], which is crucial for adequate reporting of symptoms and adhering to preventive measures [28]. Lastly, contrary to patients admitted to general hospital departments, admitted psychiatric patients are often ambulatory, eat meals together, undergo group therapy and interact with one another in close proximity, consequently facilitating the rapid spread of infection. The aforementioned aspects cause psychiatric departments to be at a higher risk for nosocomial outbreaks, thereby reinforcing the notion that the psychiatric population should be considered a high risk group. Nonconventional methods of infection prevention such as having patients use personal protective equipment (PPE) instead of isolation, which were successfully implemented in a French psychiatric hospital during previous influenza outbreaks, should be considered during outbreak management on psychiatric departments in the future [28]. Additionally, more proactive testing, especially when in doubt or when patients are not able to adequately communicate symptoms, broadening disease assessment tools and choosing to isolate patients sooner, should also be considered.

### Healthcare workers

Social distancing measures, PPE guidelines and infection prevention policies were not adequately followed by our staff. This resulted in two possible clusters of transmission. The first one took place between nurses and patients on the psychiatric ward, whilst the second one occurred between junior doctors working in close proximity to one another in the consultation-liaison office.

Factors that contributed to a lower adherence to the implemented precautionary measures amongst our HCWs are thought to be a lack of urgency, unfamiliarity with infection control measures and aspects typically associated with the mental health care system, such as frequent interdisciplinary meetings, group therapy and prolonged interactions with psychiatric patients, often taking place in close proximity. Another contributing factor might be that HCWs often continue to work with mild symptoms, and may potentially infect others while doing so, thereby facilitating and maintaining a nosocomial outbreak [29, 30].

The outbreak might have been limited if social distancing measures, PPE guidelines and infection prevention policies had been followed satisfactorily. Additional methods of protecting HCWs include extending screening criteria for symptoms and lowering the threshold for testing. Retrospectively, it is highly recommended that HCWs are tested sooner and more frequently.

### Infrastructure

As the hospital’s infection control unit did not classify the psychiatric department as a high risk department, they were not consulted during recent renovations. Consequently, we were able to identify a number of constructional shortcomings in the departmental layout which might have made our department more susceptible to the outbreak. For instance, five out of our thirteen patients’ rooms are two person rooms with one shared shower. Additionally, only three patient rooms have their own toilets. Furthermore, the ward is accessible via four entrances and experiences a lot of intermingling of patients, visitors and personnel. Another constructional flaw might have been the layout of the liaison psychiatry office and the outpatient clinic. Five work stations are located in an office of approximately 16m^2^, meaning that social distancing policies could not be followed. In combination with insufficient ventilation and a high entry and exit rate of HCWs, it is possible that a cluster of transmissions amongst HCWs took place in this small office. Therefore, for future building renovations, it is highly recommended that the hospital infection control department is consulted in order to ensure a safe working environment for all employees and patients during infectious outbreaks, especially as there are concerns COVID-19 might become a recurring seasonal affliction [1].

### Policy

Rapidly developing insights into SARS-CoV-2 and COVID-19 made outbreak management strategies change frequently during the early stages of the pandemic, and especially for departments not used to dealing with infectious diseases, the quick succession of policy changes was difficult to follow and implement adequately. Consequently, due to errors in communication between policymakers, managers, hospital staff, OHS and the hospital infection control department, misunderstandings arose. In addition, the OHS, responsible for screening hospital staff, gave conflicting advice to employees reporting symptoms. For example, some of our employees reported symptoms and were not tested and allowed to continue working, whereas other employees, with the same complaints, were tested and received a work ban. The aforementioned makes it clear that it is of vital importance that a clear governance structure is in place to implement and execute outbreak management strategies in a time of crisis.

The medical departments in our hospital are managed by both a care manager, responsible for business administration, and a medical manager, who is responsible for medical policy and represents the department within the hospital. Due to unforeseen circumstances, our department was without a medical manager when the pandemic reached our hospital. The absence of an outspoken voice in support of our department might have led to an insufficient representation of our patients’ and HCWs’ interests during the Outbreak Management Team’s meetings.

### Limitations

This investigation has several limitations. Firstly, the identification of cases relied on self-reporting of symptoms. In order to increase our investigation’s reliability, additional symptoms were retrospectively assessed by carrying out questionnaires. Secondly, asymptomatic HCWs and patients were not tested, thereby possibly contributing to further transmission [31]. Thirdly, the identification of a potential second cluster by WGS was not possible, since not all samples contained sufficient material. Fourthly, we were not able to certainly identify the index case (patient or HCW), as SARS-CoV-2 was already widespread in the general population at the time. Lastly, WGS enabled us to discriminate between nosocomial and community acquired infections. This revealed that one HCW likely acquired the infection outside the hospital or from a patient or HCW that was not sequenced. The other sequences clustered together, which indicates a transmission cluster. However, these sequences also clustered together with other sequences from different parts of the Netherlands, indicating that the confirmed cases could have contracted the infection outside the hospital. Particularly in the beginning of an outbreak of a novel viral pathogen, it is important to realize that its genetic variation is limited, and therefore it is crucial to relate the generated sequence data to epidemiological observations.

## Conclusions

In conclusion, infection control measures designed for general hospital departments are not directly applicable to psychiatric departments. Psychiatric patients should be considered a high-risk group for infectious disease, particularly during a pandemic. Therefore, general infection prevention and outbreak management policies should be adjusted to fit the specific needs of the mental health care system. Furthermore, additional guidance should be given to psychiatric HCWs during an outbreak, as they are usually less familiar with infection prevention strategies. Finally, unambiguous communication by policymakers and a clear governance structure are pivotal for the correct implementation of infection prevention measures.

## Supplementary information

**Additional file 1**. Minimum spanning tree of the SARS-CoV-2 sequenced material.

## Data Availability

The datasets used and/or analyzed during the current study are available from the corresponding author on reasonable request.
