# Supplementary figures and images for: Why psychiatry is different - challenges and difficulties in managing a nosocomial outbreak of coronavirus disease (COVID-19) in hospital care
Source: Antimicrob Resist Infect Control. 2020 Dec 1;9:190. doi: 10.1186/s13756-020-00853-z (PMC7705849; doi:10.1186/s13756-020-00853-z)

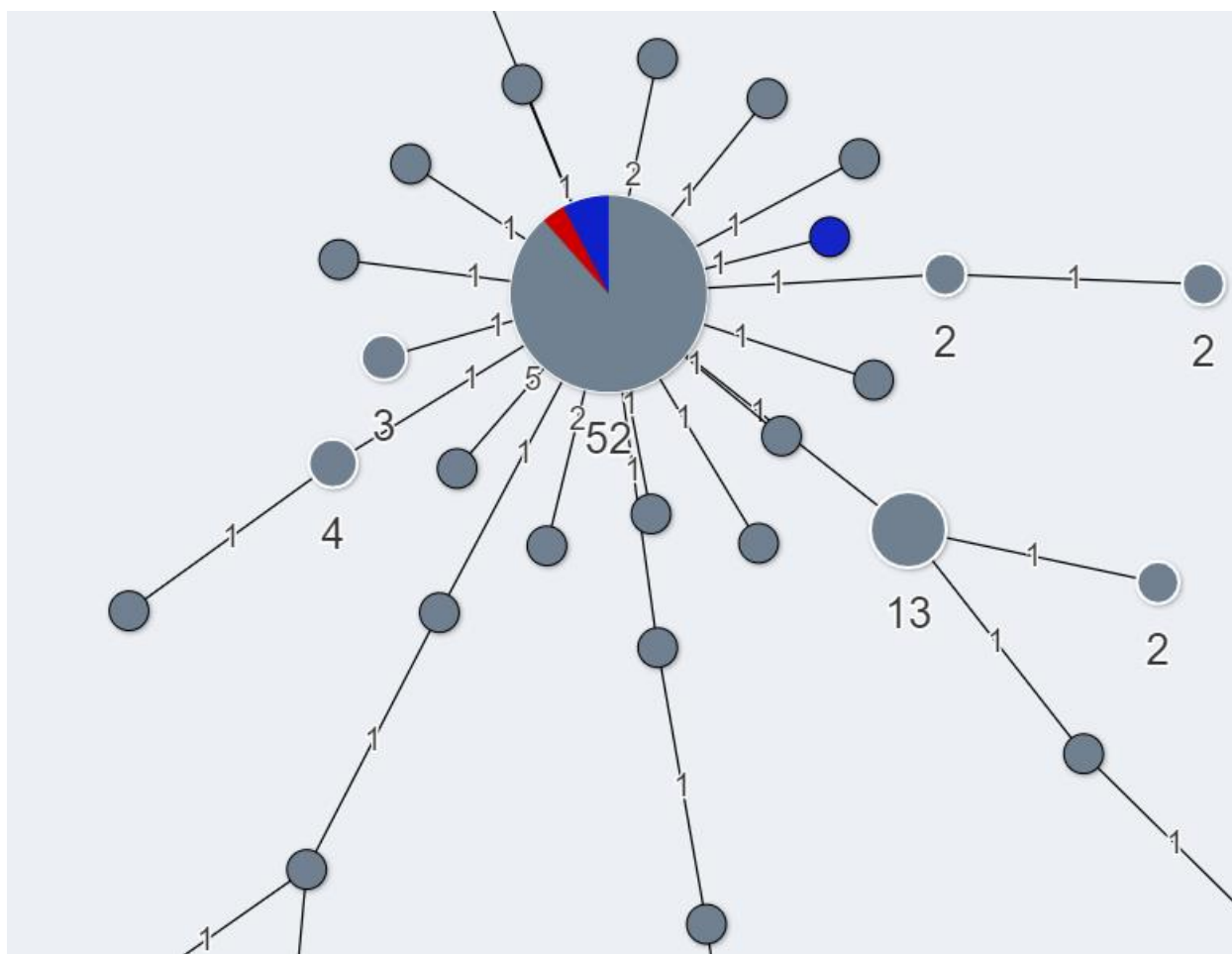

Supplement: Supplementary file 1 — Additional file 1. Minimum spanning tree of the SARS-CoV-2 sequenced material. [file 13756_2020_853_MOESM1_ESM.pdf]
